# Comparative transcriptome analyses reveal genes associated with SARS-CoV-2 infection of human lung epithelial cells

**DOI:** 10.1038/s41598-021-95733-w

**Published:** 2021-08-10

**Authors:** Darshan S. Chandrashekar, Mohammad Athar, Upender Manne, Sooryanarayana Varambally

**Affiliations:** 1 Molecular and Cellular Pathology, Department of Pathology, Wallace Tumor Institute, University of Alabama at Birmingham, 4th Floor, 20B, Birmingham, AL 35233 USA; 2 Department of Dermatology, University of Alabama at Birmingham, Birmingham, AL USA; 3 O’Neal Comprehensive Cancer Center, University of Alabama at Birmingham, Birmingham, AL USA; 4 Informatics Institute, University of Alabama at Birmingham, Birmingham, AL USA

**Keywords:** Computational biology and bioinformatics, Immunology, Diseases

## Abstract

During 2020, understanding the molecular mechanism of SARS-CoV-2 infection (the cause of COVID-19) became a scientific priority due to the devastating effects of the COVID-19. Many researchers have studied the effect of this viral infection on lung epithelial transcriptomes and deposited data in public repositories. Comprehensive analysis of such data could pave the way for development of efficient vaccines and effective drugs. In the current study, we obtained high-throughput gene expression data associated with human lung epithelial cells infected with respiratory viruses such as SARS-CoV-2, SARS, H1N1, avian influenza, rhinovirus and Dhori, then performed comparative transcriptome analysis to identify SARS-CoV-2 exclusive genes. The analysis yielded seven SARS-CoV-2 specific genes including CSF2 [GM-CSF] (colony-stimulating factor 2) and calcium-binding proteins (such as S100A8 and S100A9), which are known to be involved in respiratory diseases. The analyses showed that genes involved in inflammation are commonly altered by infection of SARS-CoV-2 and influenza viruses. Furthermore, results of protein–protein interaction analyses were consistent with a functional role of CSF2 and S100A9 in COVID-19 disease. In conclusion, our analysis revealed cellular genes associated with SARS-CoV-2 infection of the human lung epithelium; these are potential therapeutic targets.

## Introduction

Infection of severe acute respiratory syndrome coronavirus-2 (SARS-CoV-2) is the cause of human coronavirus disease 2019 (COVID-19). The recent pandemic has caused devastation due to rapid spread of this viral infection. As a respiratory illness, the disease is readily transmitted. It also has a long incubation and can be carried asymptomatically, thus spreading through communities^1^. The COVID-19 pandemic has affected almost every country, regardless of their medical infrastructure and economic status. It has caused a healthcare crisis and created a devastating economic burden, including high unemployment, which exacerbates the effect of the disease^2^. At present, more than 70 million people from 213 countries have been infected with the virus (https://www.worldometers.info/coronavirus/). The rapid spreading of this respiratory infection has forced millions to shelter in their homes and has led to death of more than 1,580,000 individuals. Additionally, in the U.S., COVID-19 has disproportionately affected patients, particularly minorities and those with chronic problems such as hypertension, lung disease, diabetes, and immunocompromised conditions.

In the last three decades, the world has witnessed zoonotic transmission of various viruses (from animals to humans) leading to severe respiratory complications. These include H1N1, avian influenza, severe acute respiratory syndrome (SARS), and Middle East respiratory syndrome coronavirus (MERS‐CoV)^3,4^. Although infections of these viruses are often fatal, their effect is generally restricted to geographic locations such as Africa, Asia, and South America. As the world awaits a vaccine for SARS-CoV-2, efforts are being made to understand the molecular mechanisms of these infections^5–9^.

High-throughput technologies such as RNA sequencing and microarrays are useful in the detection of respiratory virus infections and in understanding their molecular effect on human lung epithelial cells^10^. Extensive data on corona virus sequences have been deposited in public repositories such as NCBI Gene Expression Omnibus (GEO) and EMBL Array Express^11,12^. Meta-analysis and mining of such data can aid in a) understanding the molecular impact of COVID-19, b) elucidating differences and similarities between SARS-CoV-2 and other respiratory virus infections, and c) identifying targets for drug development. In the current study, we performed comparative analysis of publicly available gene expression data related to human lung epithelial cells infected with a respiratory virus. The analyses identified genes specifically expressed by SARS-CoV-2 infections and those that are commonly altered due to infection of coronovirus-2 and/or other respiratory viruses. In particular, expression of CSF2 (colony-stimulating factor 2) appears to be involved in COVID-19 disease. Several COVID-19 clinical trials are currently focusing on inhibition of CSF2 [GM-CSF]^13–15^.

## Results

### RNA sequencing identified genes altered on SARS-CoV-2 infection of normal human lung epithelial cells

**Figure 1:**
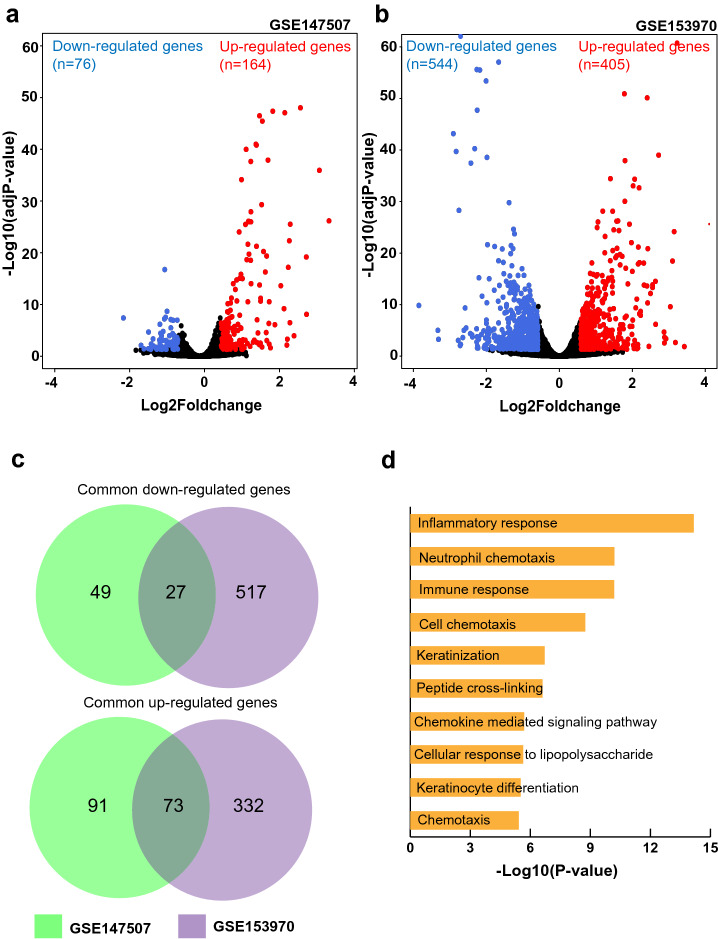
High throughput gene expression analysis of SARS-CoV-2 infection in human lung epithelial cells. **(a,b)** Volcano plots showing the proportion of differentially expressed genes on SARS-CoV-2 infection in human lung epithelial cells [from GSE147507 and GSE153970, respectively]. **(c)** Venn diagram depicting genes commonly up-/down-regulated as per both studies. **(d)** Top 10 biological processes associated with common differentially expressed genes on SARS-CoV-2 infection of human lung epithelial cells.

Differential expression analysis was conducted separately using RNA-seq data from GSE147507 and GSE153970. In total, 164 genes were up-regulated, and 76 genes were down-regulated on SARS-CoV-2 infection of normal human bronchial epithelial cells [GSE147507] (Fig. 1a). Similarly, 405 genes were up-regulated and 544 genes were down-regulated in SARS-CoV-2-infected human airway epithelium [GSE153970] (Fig. 1b). Gene list comparison identified 27 commonly down-regulated and 73 commonly up-regulated genes (Fig. 1c). Gene Ontology enrichment analysis of common differentially expressed genes showed “Inflammatory response”, “Neutrophil chemotaxis”, “Immune response”, “Cell chemotaxis”, and “Keratinization” as the top 5 enriched biological processes (Fig. 1d).

### The impact of other viral infections on the human lung epithelial cell transcriptome was explored using publicly available microarray data

**Figure 2:**
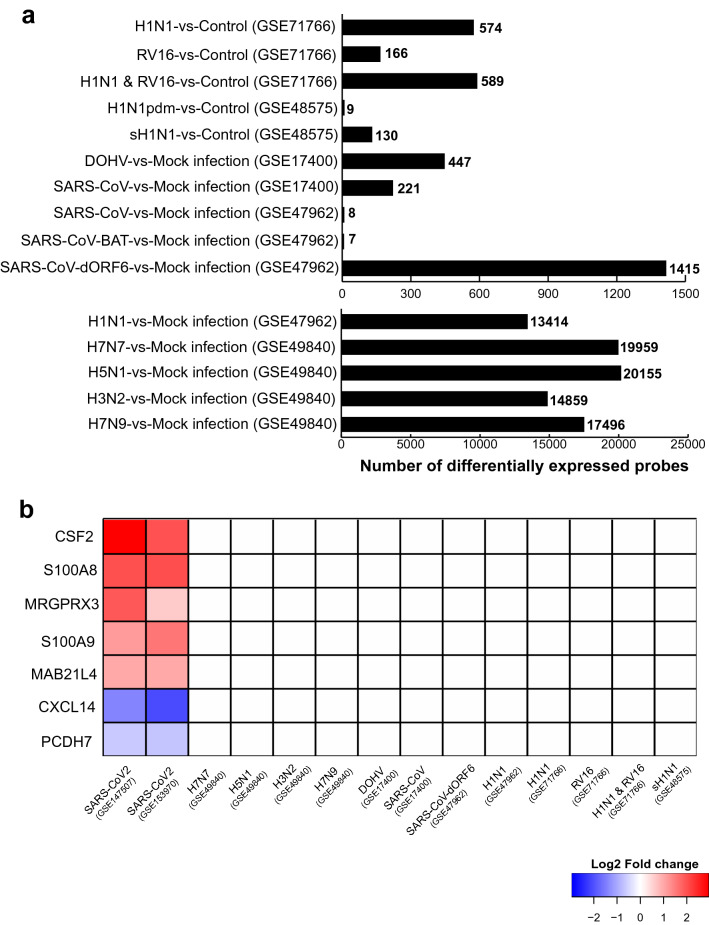
Comparative transcriptome analysis of collected gene expression data. **(a)** Histogram showing the differentially expressed genes as determined by GEO2R analysis of respiratory viral infection microarray data. **(b)** Heatmap showing 7 genes exclusively altered on SARS-CoV-2 infection.

A search of microarray datasets in the NCBI GEO database led to identification of five studies involving human lung epithelial cells subjected to viral infection. GEO2R analyses of each study were performed separately to identify differentially expressed genes. Figure 2a shows differentially expressed probes identified from each comparative analysis. From GSE71766, there were 574 differentially expressed probes (281 genes) in H1N1-infected BEAS-2B cells compared to control; 166 probes (72 genes) were up-regulated by RV16 infection. The combined infection of RV16 and H1N1 altered expression of 589 probes (288 genes). In the case of GSE49840, there were 19,959 probes (14,892 genes) differentially expressed in H7N7-infected Calu-3 cells compared to mock infected cells; 20,155 (14,948 genes), 14,859 (11,362 genes), and 17,496 probes (13,012 genes) were differentially expressed by H5N1, H3N2, or H7N9 infections, respectively. For GSE17400, DOHV or SARS-CoV infection of Calu-3 cells led to altered expression of 447 (344 genes) and 221 probes (182 genes), respectively. Analysis of GSE48575 led to identification of 130 (99 genes) and 9 (7 genes) differentially expressed probes on seasonal (sH1N1) or pandemic (H1N1pdm) influenza virus infection, respectively, of NHBECs. Lastly, the processing of GSE47962 resulted in discovery of 13,414 (7938 genes), 1415 (997 genes), 7 (5 genes), and 8 (6 genes) differentially expressed probes after H1N1, SARS-CoV-dORF6, SARS-CoV-BAT, or SARS-CoV viral infections, respectively, of HAE cells.

### Comparative analysis of DEGs resulted in identification of SARS-CoV-2 infection-specific genes and those commonly affected by most of the viral infections

In order to identify genes that are exclusively affected on SARS-CoV-2 infection, we compared common DEGs (98 protein-coding genes of 100) from RNA-seq studies with GEO2R analysis results of GSE47962, GSE17400, GSE48575, GSE49840, and GSE71766 (Supplementary Table S1). GEO2R results from each microarray study include lists of differentially expressed probes, gene symbols, fold change, and adjusted p-values. If multiple probes related to the same gene were differentially expressed, we considered the probe with highest fold change value. Comparative analysis showed 7 genes exclusively altered on SARS-CoV-2 infection, including 5 up-regulated genes (CSF2, S100A8, MRGPRX3, S100A9 and MAB21L4) and two down-regulated genes (CXCL14 and PCDH7) (Fig. 2b). However, microarray platforms showed the absence of probes related to MAB21L4 in all microarray studies (Table 1).

**Table 1:** List of probes associated with COVID-19 exclusive genes in microarray platforms related to selected gene expression studies.

Gene	Affymetrix human genome U133 plus 2.0 array	Affymetrix human genome U219 array	Agilent-014850 whole human genome microarray 4 × 44 K G4112F	Agilent-039494 SurePrint G3 human GE v2 8 × 60 K microarray	Illumina Human HT-12 V4.0 expression beadchip
CSF2	210228_at 210229_s_at	11728876_at	A_23_P133408	A_23_P133408	ILMN_1661861
S100A9	203535_at	11716523_at	A_23_P23048	A_23_P23048	ILMN_1750974
S100A8	214370_at	11753823_a_at	A_23_P434809	A_23_P434809	ILMN_1729801
MAB21L4					
MRGPRX3	1553293_at	11738052_at	A_23_P389371		ILMN_1773546
CXCL14	218002_s_at	11756059_a_at 11717910_at 11717911_x_at 11717912_s_at	A_23_P213745		ILMN_1748323
PCDH7	228640_at 205534_at 205535_s_at	11737384_at 11741722_at 11724981_at 11724982_s_at 11724983_at	A_23_P212888 A_23_P378364 A_24_P914638 A_23_P310921	A_33_P3509019	ILMN_1766668

Inflammation-related genes (such as IL6, IL1A, IL1B, CXCL2, CXCL6, CCL20, TNIP1, VNN1, TNFAIP3 and NFKBIZ) were commonly affected due to infection of SARS-CoV-2 and other SARS or avian/human influenza viruses (Supplementary Figure S1c).

### Validation of SARS-CoV-2 exclusive genes in human bronchial organoid RNA-seq data

Processing and analysis of RNA-seq data related to SARS-CoV-2-infected human bronchial organoids (HBO) led to identification of 1532 differential expressed genes (861 up-regulated and 671 down-regulated genes) (Supplementary Figure S1a). With the exception of MAB21L4, all SARS-CoV-2 exclusive genes from comparative transcriptome analysis showed the same expression pattern in HBO cells (Supplementary Figure S1b).

### Protein–protein interaction analysis of altered genes on SARS-CoV-2 infection revealed hub genes

**Figure 3:**
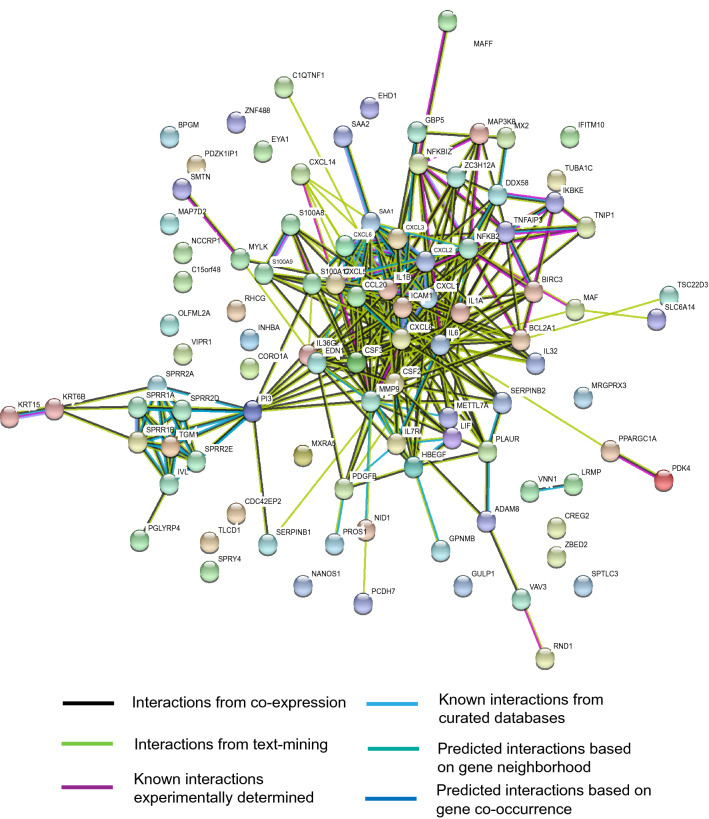
Protein–protein interaction network of genes altered by SARS-CoV-2 infection. The network with 333 edges among 72 nodes was obtained from the STRING database. Interactions with scores of ≥ 0.4 were considered.

**Figure 4:**
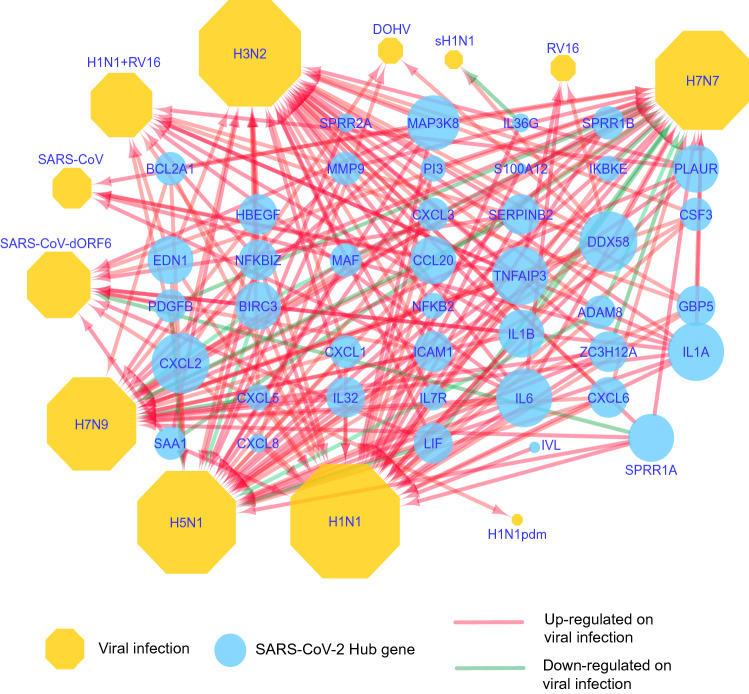
Association of COVID 19 hub genes with other respiratory viral infections. A network generated by use of Cytoscape depicts the expression of COVID-19 hub genes on infection by various respiratory viruses. The green arrows indicate down-regulation, and the red arrows show up-regulation of these genes.

The common 98 protein-coding, differentially expressed genes on SARS-CoV-2 infection were first queried in the STRING database to identify known/predicted interactions among them. The database returned a PPI network of 333 interactions (edges) between 72 genes (nodes) (Fig. 3). The PPI network was downloaded as a simple interaction format (SIF) file, visualized with Cytoscape, and analyzed with Cytohubba plugin to identify hub genes. The top 50 genes were obtained based on three network parameters: degree, closeness, and betweenness separately. The 43 genes featured in all three lists were considered as hub genes (Table 2). The hub genes were IL6, IL1B, CXCL8, MMP9, CXCL1, CSF2, CCL20, ICAM1, IL1A, CXCL2, CSF3, SAA1, NFKB2, PI3, TNFAIP3, CXCL3, CXCL5, CXCL6, EDN1, HBEGF, BCL2A1, NFKBIZ, S100A12, PLAUR, BIRC3, IL36G, LIF, SERPINB2, SPRR1A, SPRR1B, IVL, DDX58, SPRR2A, ZC3H12A, S100A9, PDGFB, IL7R, MAP3K8, IKBKE, MAF, ADAM8 and GBP5. Among hub genes, most were affected by infection of human or avian influenza viruses such as H7N7, H1N1, H7N9, H3N2, and H5N1 (Fig. 4); CSF2 and S100A9 were exclusive to SARS-CoV-2 infection.

**Table 2:** Hub genes involved in SARS-CoV-2 infection of normal human bronchial epithelial cells.

Hub nodes	Degree	Closeness	Betweenness	GSE147507		GSE153970	
				Absolute FC	P-value	Absolute FC	P-value
IL6	39	52.66	1061.909	7.59	6.59E−20	5.87	4.78E−14
IL1B	35	50.16	715.3514	2.11	9.62E−25	1.67	5.66E−11
CXCL8	34	49.66	382.1511	5.28	2.6E−112	2.38	5.98E−24
MMP9	30	47.91	832.9246	5.50	4.86E−23	2.41	1.51E−14
CXCL1	26	43.95	143.7859	2.64	2.27E−38	2.63	3.04E−19
CSF2	23	43.83	194.193	7.63	8.01E−09	3.97	6.06E−05
CCL20	22	43.16	99.528	9.31	1.37E−71	3.44	1.25E−51
ICAM1	21	42.83	130.012	3.66	1.24E−38	1.53	6.44E−05
IL1A	19	40.28	23.204	2.14	8.95E−16	1.71	5.09E−10
CXCL2	19	40.45	39.909	2.65	9.33E−15	2.15	1.75E−09
CSF3	18	39.61	18.700	32.18	7.74E−18	9.36	1.77E−61
SAA1	17	38.95	142.325	5.04	8.62E−48	1.56	1.53E−06
NFKB2	17	39.61	122.589	2.01	1.82E−15	1.78	3.29E−08
PI3	17	40.75	1217.573	3.72	4.64E−07	1.93	2.59E−07
TNFAIP3	17	38.56	46.582	3.11	3.38E−47	1.73	1.11E−08
CXCL3	17	39.28	22.292	4.67	2.38E−14	3.67	9.64E−15
CXCL5	15	38.11	8.458	11.70	6.87E−27	4.18	4.61E−35
CXCL6	14	37.61	7.723	3.26	0.016423	2.74	5.63E−13
EDN1	13	39	122.702	2.13	1.19E−08	1.88	1.94E−10
HBEGF	11	35.95	144.650	2.52	8.02E−27	2.75	2.78E−15
BCL2A1	11	36.11	139.405	4.97	6.48E−10	3.30	4.38E−20
NFKBIZ	11	35.4	139.988	1.82	5.76E−12	2.28	7.37E−29
S100A12	11	37.5	25.471	2.21	0.0438	5.28	2.45E−14
PLAUR	9	35.2	74.008	1.65	2.5E−06	2.25	2.30E−16
BIRC3	9	34.53	8.979	3.36	5.7E−21	1.60	2.55E−05
IL36G	9	34.95	0.821	6.79	9.54E−49	14.64	1.43E−88
LIF	9	34.56	3.828	2.52	1.05E−26	2.60	8.75E−10
SERPINB2	8	34.45	0.689	1.68	4.64E−07	4.55	2.23E−33
SPRR1A	8	29.28	86.666	1.67	0.00927	5.88	1.43E−14
SPRR1B	8	29.28	86.666	1.53	0.001373	3.48	1.15E−38
IVL	8	29.11	136	2.02	2.08E−11	2.97	3.49E−20
DDX58	8	33.9	109	1.50	0.0088	1.55	2.54E−05
SPRR2A	8	29.28	86.666	3.57	4.3E−20	18.08	1.04E−152
ZC3H12A	7	33.56	4.0976	3.24	5.01E−30	1.92	3.20E−12
S100A9	7	33.78	0.333	2.21	7.38E−35	2.97	6.39E−27
PDGFB	7	33.78	138.966	2.06	6.93E−07	1.95	3.74E−08
IL7R	7	33.4	4.91	1.83	0.013	2.48	0.0414
MAP3K8	6	32.73	0.44	2.22	0.005	2.25	1.96E−11
IKBKE	6	31.98	3.31	1.71	0.00017	1.68	9.13E−07
MAF	3	30.48	136	− 1.73	3.26E−06	− 1.81	0.00755
ADAM8	3	29.11	268	2.18	3.26E−06	2.33	3.83E−15
GBP5	3	29.73	31.94	3.84	0.02372	4.26	4.21E−10

## Discussion

Microarray meta-analysis and comparative transcriptome analysis have been useful bioinformatic approaches for maximum utilization of publicly available gene expression data^16,17^. Since the advent of high-throughput technologies such as microarrays and RNA-seq, researchers have performed in-depth transcriptome analyses of various biological conditions, leading to various discoveries^18^. Data from thousands of such experiments are being deposited in public repositories such as GEO and ArrayExpress. Selection and combinatorial analysis of such data can aid researchers in understanding the molecular mechanisms of a disease and in discovering biomarkers^19–21^. We observed the availability of various microarray studies related to human respiratory viral infection and sensed the opportunity to compare the effect of SARS-CoV-2 and other respiratory viral infections on the human lung transcriptome.

The comparative transcriptome analysis led to identification of genes that were altered exclusively after SARS-CoV-2 infection. Among these genes, S100 calcium-binding protein A9 (S100A9) and S100 calcium-binding protein A8 (S100A8) are calcium- and zinc-binding proteins that are elevated in inflammatory lung disorders^22^. S100A8 and S100A9 form a heterodimer complex called Calprotectin (CLP). Elevated levels of CLP is found in bronchoalveolar lavage fluid (BALF), serum and lung tissue of pneumonia patients^23^. Serum CLP could be potential biomarker for COVID-19 and further research is required to test this hypothesis^24^. Colony stimulating factor 2 (CSF2) is a cytokine-coding gene associated with respiratory diseases such as pulmonary alveolar proteinosis^25^. CSF2 (GM-CSF) is known to be pro-inflammatory cytokine produced by wide variety of cells such as macrophages, T-cells, fibroblast, tumor cells, endothelial cells, and with primary production at the inflammation site^26,27^. GM-CSF influences activation and proliferation of immune cells such as macrophages, monocytes, dendritic cells, neutrophils, eosinophils^28^. Although GM-CSF plays important role in maintaining immune homeostasis, its over-expression in lung could lead to fibrotic reactions and severe immune cell infiltrations^29^. Recent reports show that a) COVID-19 patients requiring intensive care unit (ICU) have increased level of GM-CSF in China and b) drugs targeting CSF2 (GM-CSF) or its receptor (such as lenzilumab, namilumab, gimsilumab, and otilimab) are being evaluated in clinical trials with COVID-19 patients^13–15,30^. MAS-related GPR family member X3 (MRGPRX3), a member of the mas-related/sensory neuron specific subfamily of G protein coupled receptors, is down-regulated in human airway epithelial cells exposed to smoke from electronic cigarettes^31^. In oral cancers, lung cancers, and head and neck cancers, C-X-C Motif chemokine ligand 14 (CXCL14) functions as a tumor suppressor; it also induces growth of prostate and breast cancers^32–36^. Protocadherin 7 (PCDH7) is involved in cell–cell recognition and adhesion^37^. Mab-21-like 4 (MAB21L4) has no known association with lung disorders or respiratory virus infections.

The PPI analysis of genes differentially expressed by SARS-CoV-2 infection identified 43 hub genes. CSF2 and S100A9 were the only hub genes to show a SARS-CoV-2-exclusive gene expression pattern. Almost all other hub genes were affected by infection of other respiratory viruses. In conclusion, both PPI analysis and comparative transcriptome analysis point to a role of *CSF2* in the molecular mechanism of SARS-CoV-2 infections of human lung epithelium. The current study also highlights the exclusivity of known lung inflammation disorder genes such as S100A8 and S100A9 with respect to SARS-CoV-2 infection.

Our current study utilizes exclusively the transcriptomic data. Similar comparative analysis as well as integrative analysis with proteomics and other “omic” data will be useful as more data becomes public. The current bioinformatics approach makes use of available data to identify potential molecular targets for treatment of COVID-19. Future studies will focus on experimental validation to establish the exclusive association of CSF2, S100A8 and S100A9 with SARS-CoV-2 infections in lung epithelial cells.

## Methods

### RNA sequencing data analysis

The NCBI GEO database was searched for microarray or RNA-sequencing data related to SARS-CoV-2 infections of human lung epithelial cells. We evaluated two studies, GSE147507 and GSE153970^6,38^. GSE147507 included normal human bronchial epithelial cells subjected to mock treatment (n = 3) or to SARS-CoV-2 infection (n = 3); GSE153970 included primary human airway epithelial cultures infected with mock (n = 3) or SARS-CoV-2 (n = 3). For the purpose of validation, we also selected RNA-seq data related to SARS-CoV-2-infected human bronchial organoids [GSE150819].

Raw sequencing data related to selected samples were downloaded from Sequence Read Archive (SRA) using fastq-dump of sratoolkit v2.9.6 (http://ncbi.github.io/sra-tools/). First, raw sequencing reads were trimmed to remove adapter sequences and low-quality regions using Trim Galore! (v0.4.1) (http://www.bioinformatics.babraham.ac.uk/projects/trim_galore/). Trimmed reads were subjected to quality control analysis using FastQC (https://www.bioinformatics.babraham.ac.uk/projects/fastqc/). Tophat v2.1 was used to map trimmed raw reads to the human reference genome (hg38)^39^. All bam files from multiple runs related to the same samples were merged and sorted using SAMtools (Version: 1.3.1)^40^. Finally, raw read counts were enumerated for each gene in each sample using GTF (gene transfer file) from Ensembl [Homo.sapiens_GRCh38.82.gtf] and HTSeq-count^41^.

Analysis of differential expression was performed using DESeq2 according to a standard protocol (https://bioconductor.org/packages/release/bioc/vignettes/DESeq2/inst/doc/DESeq2.html)^42^. Genes with adj.P-value < 0.05 and absolute fold change ≥ 1.5 were considered as significantly differentially expressed. Common up-regulated and down-regulated genes from GSE147507 and GSE153970 were obtained using the Venny online tool (https://bioinfogp.cnb.csic.es/tools/venny/).

Gene ontology enrichment analyses of the common Differentially Expressed Genes (DEGs) were accomplished by use of the Database for Annotation, Visualization and Integrated Discovery (DAVID) v6.8 online tool^43^. Gene ontology (GO) biological processes with P-values < 0.05 and gene counts > 2 were considered as significantly enriched.

### Microarray data collection and analysis

The NCBI GEO database was queried for microarray data related to SARS-CoV infections of human lung epithelial cells. A query *(SARS-CoV) AND "Homo sapiens"[porgn] AND ("gse"[Filter] AND ("Expression profiling by array"[Filter]))* led to 15 search results. After screening, two studies (GSE47962, GSE17400) were selected. To find microarray data related to SARS-CoV infections of human lung epithelial cells, the GEO database was queried using *(((Human lung epithelium) OR (Human bronchial epithelial) AND "Homo sapiens"[porgn] AND ("gse"[Filter] AND "Expression profiling by array"[Filter]))) AND (viral infection AND ("gse"[Filter] AND "Expression profiling by array"[Filter])) AND ("gse"[Filter] AND "Expression profiling by array"[Filter]) AND ("Expression profiling by array"[Filter])*. This led to 38 search results, three of which (GSE49840, GSE71766, and GSE48575) were selected for analysis. Table 3 provides sample, platform, and cell line details for all five studies.

**Table 3:** Summary of publicly available, high-throughput experimental data considered in the current study.

*GEO accession number*	Cell line	Virus infection studied after 24 h (along with GSM ids)	Platform considered
*GSE147507*	Normal Human Bronchial Epithelial (NHBE)	**SARS-CoV-2** (*GSM4432381, GSM4432382, GSM4432383*), **Mock treatment** (*GSM4432378, GSM4432379, GSM4432380*)	Illumina NextSeq 500 (*Homo sapiens*)
*GSE153970*	Human airway epithelium (HAE)	**SARS-CoV-2** (*GSM4661083, GSM4661084, GSM4661085*)**, Mock treatment** (*GSM4661080, GSM4661081, GSM4661082*)	Illumina NovaSeq 6000 (*Homo sapiens*)
*GSE47962*	Human airway epithelium (HAE)	**SARS-CoV** (*GSM1163617, GSM1163618, GSM1163619*), **H1N1** (*GSM1163602, GSM1163603, GSM1163604*), **SARS-CoV-BatSRBD** (*GSM1163543,GSM1163544*), **SARS-CoV-dORF6** (*GSM1163569, GSM1163570, GSM1163571*), **Mock treatment** (*GSM1163650,GSM1163651, GSM1163652*)	Agilent-014850 whole human genome microarray 4 × 44 K G4112F
*GSE49840*	Polarized Calu3	**H7N7** (*GSM1208108, GSM1208109, GSM1208110, GSM1208111*), **H5N1** (*GSM1208139, GSM1208140, GSM1208141, GSM1208142*), **H3N2** (*GSM1208124, GSM1208125, GSM1208126, GSM1208127*), **H7N9** (*GSM1208076, GSM1208077, GSM1208078, GSM1208079*), **Mock treatment** (*GSM1208092, GSM1208093, GSM1208094, GSM1208095*)	Agilent-039494 SurePrint G3 human GE v2 8 × 60 K microarray
*GSE17400*	Calu3 subclone 2B4	**SARS-CoV** (*GSM432332, GSM432333, GSM432360*), **DOHV** (*GSM432398, GSM432399, GSM432400*), **Mock treatment** (*GSM432033, GSM432034, GSM432209*)	Affymetrix human genome U133 Plus 2.0 array
*GSE71766*	Human bronchial epithelial (BEAS-2B)	**H1N1** (*GSM1844862, GSM1844863, GSM1844864, GSM1844865, GSM1844866*), **RV16** (*GSM1844907, GSM1844908, GSM1844909, GSM1844910, GSM1844911*), **Control** (*GSM1844817, GSM1844819, GSM1844820, GSM1844818, GSM1844821*), **H1N1 + RV16** (*GSM1844952, GSM1844953, GSM1844954, GSM1844955, GSM1844956*)	Affymetrix human genome U219 array
*GSE48575*	Normal human bronchial epithelial cells (NHBECs)	**H1N1pdm** (*GSM1181433, GSM1181434, GSM1181435*), **sH1N1** (*GSM1181442, GSM1181443, GSM118144*), **Control for H1N1pdm** (*GSM1181427, GSM1181428, GSM1181429*), **Control for sH1N1**(*GSM1181436, GSM1181437, GSM1181438*)	Illumina Human HT-12 V4.0 expression beadchip
*GSE150819*	Human bronchial organoids	**Uninfected human bronchial organoid** (*GSM4559193, GSM4559194, GSM4559195*), **SARS-CoV-2 infected human bronchial organoid** (*GSM4559196, GSM4559197, GSM4559198*)	Illumina NovaSeq 6000

Since SARS-CoV-2 RNA-seq data included transcriptome profiling after 24 h of infection, in all microarray studies, we considered only samples after 24 h of viral infection.

GSE47962 included samples from human airway epithelium (HAE) cells infected with SARS-CoV, influenza virus (H1N1), or variants of SARS-CoV (SARS-dORF6 and SARS-BatSRBD)^44^. GSE71766 comprised human bronchial epithelial cells (BEAS-2B) infected with rhino virus (RV), influenza virus (H1N1), or both (RV + H1N1)^45^. Bronchial epithelial cell line 2B4 (a clonal derivative of Calu-3 cells) infected with SARS-CoV or Dhori virus (DOHV) was part of GSE17400^46^. GSE49840 included polarized calu-3 (cultured human airway epithelial cells) infected with human influenza virus (H3N2) or avian influenza viruses (H7N9, H5N1, and H7N7)^47^. GSE48575 consisted of normal human bronchial epithelial cells (NHBEC) infected with seasonal H1N1 influenza A (sH1N1) or pandemic H1N1 influenza A (H1N1pdm)^48^. GEO2R was used to identify differentially expressed genes for each of these studies independently^12^. Probes with adj. P-value < 0.05 and absolute fold change ≥ 1.5 were considered as statistically significant and were compared with DEGs of SARS-CoV-2 infection from RNA-seq data.

### Protein–protein interaction analysis

STRING, a database of known or predicted protein–protein interactions (PPIs) was used to obtain interactions between genes altered on SARS-CoV-2 infection^49^. Output from the STRING database was uploaded to Cytoscape v3.7.2 in simple interaction format, and the Cytohubba app was employed to identify hub genes^50–52^. The top 50 genes were obtained separately from the PPI network based on three network parameters (closeness, degree, and betweenness) then common genes among these were selected as hub genes. We also checked the DrugBank database to determine if a drug is available to target them^53^.

## Supplementary Information

Supplementary Figure S1.

Supplementary Table S1.
