# Supplementary material for: Comparative transcriptome analyses reveal genes associated with SARS-CoV-2 infection of human lung epithelial cells
Source: Sci Rep. 2021 Aug 10;11:16212. doi: 10.1038/s41598-021-95733-w (PMC8355180; doi:10.1038/s41598-021-95733-w)

Comparative transcriptome analyses reveal genes associated with SARS-CoV-2 infection of human lung epithelial cells

Darshan S. Chandrashekar[1,*], Mohammad Athar[2,3], Upender Manne[1,3#], Sooryanarayana Varambally[1,3,4#*]

[1]Department of Pathology, University of Alabama at Birmingham, Birmingham, AL
[2]Department of Dermatology, University of Alabama at Birmingham, Birmingham, AL
[3]O'Neal Comprehensive Cancer Center, University of Alabama at Birmingham, Birmingham, AL
[4]Informatics Institute, University of Alabama at Birmingham, Birmingham, AL

Share Senior Authorship

*Correspondence to: Sooryanarayana Varambally, Ph.D., Molecular and Cellular Pathology, Department of Pathology, Wallace Tumor Institute, 4th floor, 20B, University of Alabama at Birmingham, Birmingham, AL 35233, USA Phone: (205) 996-1654
Email: soorya@uab.edu
And
Darshan S. Chandrashekar Ph.D., Department of Pathology, University of Alabama at Birmingham, Birmingham, AL
Email: dshimogachandrasheka@uabmc.edu

Supplementary figure S1: Summarization of comparative transcriptome analysis and validation. (a) Volcano plot showing result of differential expression analysis using RNA-seq data associated with SARS-CoV-2-infected human bronchial organoids. (b) Heatmap showing expression pattern of SARS-CoV-2 exclusive genes in SARS-CoV-2-infected human bronchial organoids. (c) Genes commonly altered in human lung epithelial cells on infection of SARS-CoV-2, SARS and other human/avian influenza viruses.

Supplementary figure S1

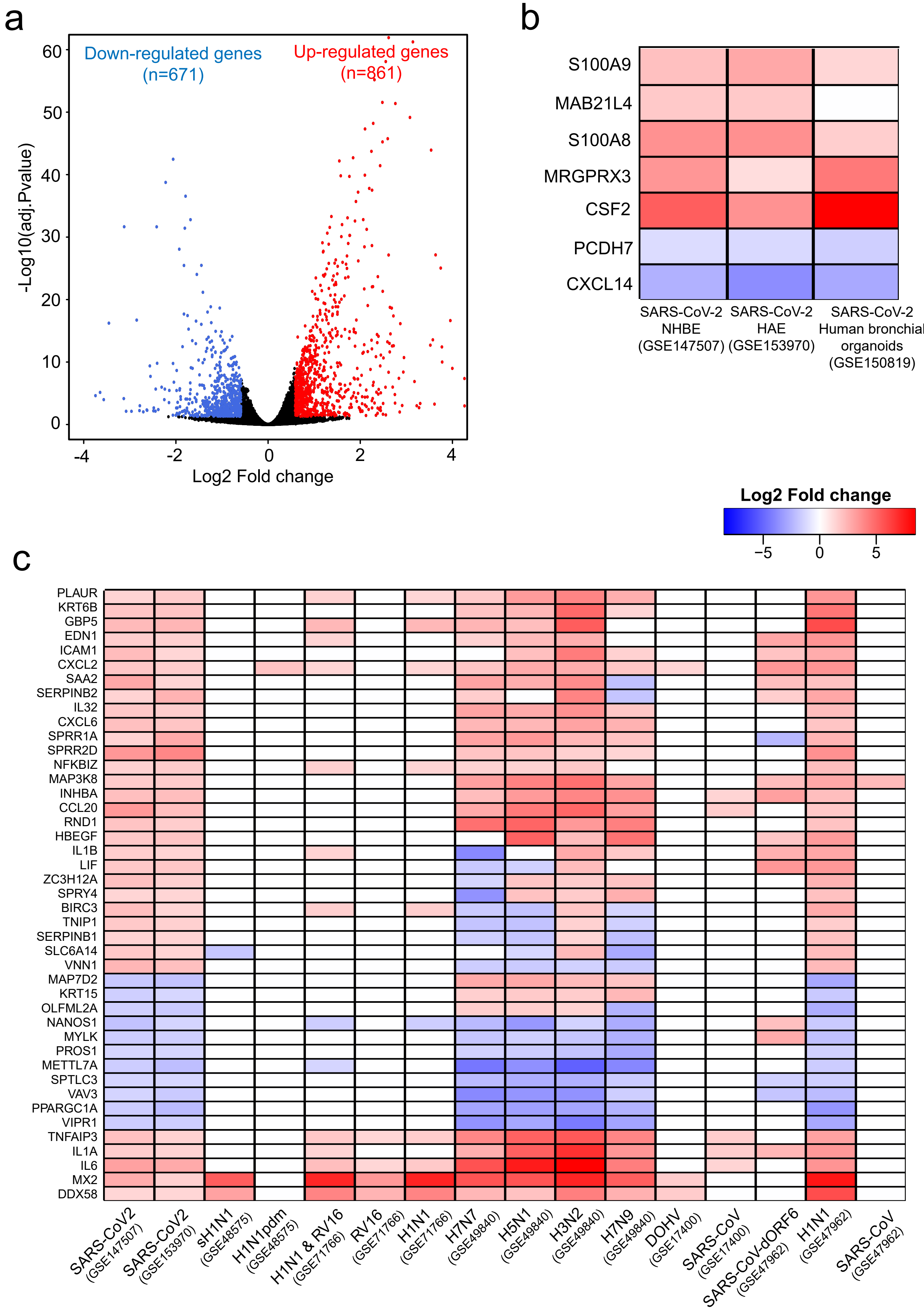

Supplement: Supplementary file 1 — Supplementary Figure S1. [file 41598_2021_95733_MOESM1_ESM.pdf]
